# The role of nitric oxide and hydrogen sulfide in regulation of redox homeostasis at extreme temperatures in plants

**DOI:** 10.3389/fpls.2023.1128439

**Published:** 2023-02-07

**Authors:** Yuriy E. Kolupaev, Alla I. Yemets, Tetiana O. Yastreb, Yaroslav B. Blume

**Affiliations:** ^1^ Yuriev Plant Production Institute, National Academy of Agrarian Sciences of Ukraine, Kharkiv, Ukraine; ^2^ Institute of Food Biotechnology and Genomics, National Academy of Sciences of Ukraine, Kyiv, Ukraine

**Keywords:** nitric oxide, hydrogen sulfide, reactive oxygen species, redox regulation, calcium, hypothermia, hyperthermia, stress-protective systems

## Abstract

Nitric oxide and hydrogen sulfide, as important signaling molecules (gasotransmitters), are involved in many functions of plant organism, including adaptation to stress factors of various natures. As redox-active molecules, NO and H_2_S are involved in redox regulation of functional activity of many proteins. They are also involved in maintaining cell redox homeostasis due to their ability to interact directly and indirectly (functionally) with ROS, thiols, and other molecules. The review considers the involvement of nitric oxide and hydrogen sulfide in plant responses to low and high temperatures. Particular attention is paid to the role of gasotransmitters interaction with other signaling mediators (in particular, with Ca^2+^ ions and ROS) in the formation of adaptive responses to extreme temperatures. Pathways of stress-induced enhancement of NO and H_2_S synthesis in plants are considered. Mechanisms of the NO and H_2_S effect on the activity of some proteins of the signaling system, as well as on the state of antioxidant and osmoprotective systems during adaptation to stress temperatures, were analyzed. Possibilities of practical use of nitric oxide and hydrogen sulfide donors as inductors of plant adaptive responses are discussed.

Temperature is a factor that plants cannot effectively control. In this regard, it is the ambient temperature that limits growth, productivity and distribution of plants in climatic zones. For example, high temperatures are now believed to be a major determinant of crop growth and yields at the global level (Ali et al., 2020). According to predictive models, the average temperature on the planet may increase by 3-6°C by the middle of this century, significantly increasing the likelihood of thermal damage to plants (Mohapatra, 2019). This challenge is especially relevant for Europe, where temperature increase trends come out three to four times faster than in the other northern latitudes (Rousi et al., 2022). However, despite the increase in the mean annual temperature in recent decades, the relevance of plant resistance to cold and frost for many countries of the world not only lessens, but becomes greater (Chervenkov and Slavov, 2022). Winter thaws alternating with sudden frosts damage plants and decrease their productivity. Winter cereals are most affected by the cold, causing the loss of their productivity.

The effects of low and high temperatures on plants at the cellular and molecular levels have both fundamental differences and significant similarities (Zhen and Ungerer, 2008). For instance, a decrease in temperature causes a very rapid increase in the rigidity of cell membranes due to phase transitions of the lipid bilayer (Kazemi-Shahandashti and Maali-Amiri, 2018). On the other hand, exposure to high temperatures leads to fluidization of the lipid part of the membranes (Li et al., 2018). At the same time, however, both types of exposure disrupt the native state of cell membranes and enhance the stochastic formation of reactive oxygen species (ROS), primarily in the inner membranes of chloroplasts and mitochondria (Asada, 1999; Han et al., 2013; Bernfur et al., 2017; Choudhury et al., 2017). Along with this, both low- and high-temperature exposures cause an increase in the ROS generation on the cell surface by NADPH oxidase (Piotrovskii et al., 2011; Kolupaev et al., 2013; Yao et al., 2017). ROS generated under stressful temperatures are considered to be components of a signaling network that ensures the formation of the adaptive plant responses to these stress factors (Zhang et al., 2016).

The action of low and high temperatures on cell membranes also leads to changes in calcium homeostasis. Opening of calcium channels is one of the earliest responses of plant cells to cooling (Jian et al., 1999). The obtained experimental data indicate the possibility of cold-induced calcium release into cytosol by several pathways: involving COLD1-RGA1 complex (Ma et al., 2015b), *via* cyclic nucleotide-gated channels (CNGCs) (Chen et al., 2021), as well as Ca^2+^-permeable transporter Annexin1 (AtANN1) (Liu et al., 2021). Changes in the cytosolic calcium concentration due to the effect of high temperatures on membranes are primarily associated with the opening of specific thermosensitive CNGCs (Saidi et al., 2009; Gao et al., 2012; Finka and Goloubinoff, 2014). Thus, changes in calcium homeostasis are considered to be a key element in the transduction of cold and heat stress signals.

Along with such universal signaling mediators as ROS and calcium ions, the main gasotransmitters, nitric oxide (NO) (Fancy et al., 2017; Yemets et al., 2019) and hydrogen sulfide (H_2_S) (Fu et al., 2013; Kolupaev et al., 2022; Raza et al., 2022), are involved in signal transduction caused by extreme temperatures. To date, experiments with plants of different taxonomic affiliations have revealed increases in the content of NO and H_2_S in response to both low (Liu et al., 2013b; Du et al., 2017; Fancy et al., 2017; Liu et al., 2019) and high (Christou et al., 2014; Karpets et al., 2015a; Chen et al., 2016) temperatures. However, the causal relationships of such effects with changes in cellular content of other signaling mediators, including such important ones as ROS, remain largely understudied. Moreover, information on the role of gasotransmitters in cold adaptation of plants is quite contradictory. Even for the best studied signaling molecule NO, both positive (Zhao et al., 2009; Puyaubert and Baudouin, 2014) and negative (Costa-Broseta et al., 2018) regulation of gene expression critical for adaptation to extreme temperatures has been reported on the same objects.

Proteomics and bioinformatics methods have identified a large number of proteins that can undergo post-translational modification by NO, H_2_S, and ROS (Valderrama et al., 2019; Corpas et al., 2022). However, this vast body of information is still poorly interpreted in the context of specific physiological processes and their role in plant adaptation to extreme temperatures. In many cases, such interpretation becomes more complicated due to parallel processes of direct interaction between redox active molecules and proteins and their mediated effect on the expression of genes important for adaptation. Thus, understanding of the mechanisms of functional interaction of NO, H_2_S, and ROS as signal mediators involved in the induction of adaptive responses to extreme temperatures is far from being fully elaborated. The analysis of data on the role of functional relationships between two key gasotransmitters (nitrogen oxide and hydrogen sulfide) and ROS in plant adaptation to low and high temperatures has been the main objective of this review. Since the effects of extreme high and low temperatures on plants are accompanied by a disruption of the water regime, we also discuss in some cases the processes of functional interaction of signaling molecules in the formation of protective responses to dehydration.

## Nitric oxide synthesis in plants at stress temperatures

### Main pathways of NO synthesis in plants

Despite intensive research that has revealed the diversity of nitric oxide functions in plants, its synthesis still remains one of the most difficult challenges in this field (Astier et al., 2018). There are two main ways of NO synthesis in plants: reductive, based on the reduction of nitrites to NO, and oxidative, related to the oxidation of molecules containing amino groups (Kumar and Ohri, 2023).

The reductive pathway is a well-described and proven pathway for nitric oxide synthesis in plants. Nitrate reductase (NR), a multifunctional cytoplasmic enzyme involved in nitrogen assimilation and metabolism, is considered one of the key enzymes for NO synthesis. It is responsible for the first limiting step in nitrate assimilation, catalyzing reduction of nitrate to nitrite using NADH or NADPH as an electron donor. Active enzymatic homodimeric complex requires the presence of molybdopterin, heme, and FAD as cofactors (Astier et al., 2018). In addition to its primary activity, NR also possessed nitrite-NO reductase activity (Ni-NR activity) (Rockel et al., 2002). Under normal conditions, this activity is only 1% of nitrate-reducing capacity of NR (Astier et al., 2018). However, factors such as an anoxic or acidic environment contribute to NO formation by NR. Despite such specific conditions, the significant contribution of NR-mediated NO production in plant physiology has been convincingly demonstrated using both pharmacological and genetic approaches (Mur et al., 2013).

A specific mechanism for NR-mediated NO formation was discovered in the case of *Chlamydomonas reinhardtii*. In this unicellular alga, NR can interact with its partner protein, the amidoxime reducing component (ARC), forming a catalytic complex with it (Chamizo-Ampudia et al., 2017). The ARC protein within such a complex was named nitric oxide-forming nitrite reductase (NOFNiR). It is the one that catalyzes NO formation from nitrite. It was shown that gene expression patterns and enzymatic activity of NR and NOFNiR correlate with each other (Chamizo-Ampudia et al., 2017). We assume that detection of such enzymatic system in higher plants will help to better explain the crucial role of NR in NO synthesis by plants (Astier et al., 2018).

Thus, the functions of NR and its partner proteins can be complex and diverse. It is notable that many plants, in particular *Arabidopsis thaliana*, *Nicotiana tabacum*, *Hordeum vulgare*, *Zea mays*, *Brassica napus*, *Glycine max*, *Oryza sativa*, have two or more NR isoforms that can perform different functions (Mohn et al., 2019). When comparing the functional properties of *A. thaliana* NR isoforms, NIA2 has been found to function in nitrate reduction process, whereas NIA1 is mainly involved in NO synthesis (Mohn et al., 2019).

Another possible reductive pathway for NO synthesis is the one associated with xanthine oxidoreductase localized in peroxisomes (Farnese et al., 2016).

In addition to the reductive pathways for NO synthesis from nitrite/nitrate listed above, several lines of evidence demonstrate the existence of an oxidative pathway for NO generation in plants similar to the one described in animals. However, to date, animal NO synthase homologues have been identified only in green algae (*Ostreococcus tauri* and *Ostreococcus lucimarinus*), but not in higher plants (Roszer, 2014; Hancock and Neill, 2019). It is assumed that the NO synthase gene (NOS) was lost during the evolution of plants (Jeandroz et al., 2016). NOS has not been detected in any of the more than 1000 screened transcriptomes of terrestrial plants (Jeandroz et al., 2016).

Nevertheless, the presence of NOS-like activity in plants has been confirmed by other methods, including those that allow direct measurement of NO generation (Chaki et al., 2009). This enzymatic activity has been termed NOS-like since it has been reported to be strictly dependent on the presence of arginine and NADPH, as well as several NOS cofactors, as in the case of the animal enzyme. This NOS-like activity has been found in chloroplasts, mitochondria, and peroxisomes (Corpas and Barroso, 2014; Farnese et al., 2016). However, clear evidence of the existence of the corresponding protein in higher plants is still lacking (Astier et al., 2018).

Recently, not only L-arginine, but also polyamines and hydroxylamine have been considered as substrates for NO formation in oxidative pathway (Wimalasekera et al., 2001; Hancock and Whiteman, 2014). It has been suggested that these transformations can be catalyzed by di- and polyamine oxidases localized mainly in cell walls (Saha et al., 2015).

Nitric oxide can also be obtained non-enzymatically from nitrite in the presence of a reducing agent such as ascorbate. Since this reaction requires an undissociated acid, it can occur in certain microenvironments under acidic conditions, in particular in apoplast, vacuole, and cellular compartments under unbalanced redox conditions (Grossi and Casadei, 2021). Another possible pathway for non-enzymatic nitric oxide production is NO released from S-nitrosoglutathione (GSNO) (Kumar and Ohri, 2023).

It should be noted that GSNO is the most significant reservoir of NO (Gupta et al., 2011). As is known, nitric oxide can react with glutathione (GSH) to form GSNO, which in turn can again serve as a NO donor in the cell. GSNO content is regulated by S-nitrosoglutathione reductase (GSNOR), which reduces GSNO to glutathione sulfinamide (GS(O)NH_2_) using NADH (Gupta et al., 2011).

### Nitric oxide synthesis in plants during adaptation to cold

An increase in NO content in response to cold exposure was found in organs of plants of different taxonomic affiliation (*Arabidopsis thaliana*, *Pisum sativum*, *Triticum aestivum*, *Citrus aurantium*) (Yemets et al., 2019). It is noteworthy that such an effect was caused by both relatively short-term (1-4 hours) and long-term (7-14 days) exposures of plants to low temperatures (Ziogas et al., 2013; Puyaubert and Baudouin, 2014; Baudouin and Jeandroz, 2015; Fancy et al., 2017). At the same time, the increase in the content of NO in the tissues was stable. For example, in Arabidopsis, a gradual increase in NO content in leaves was recorded, which reached a maximum on the 14th day of exposure at 4°C (Zhao et al., 2009).

The main enzyme of NO synthesis induced by hypothermia in plants is probably nitrate reductase. Thus, in Arabidopsis *nia1nia2* double mutants, at low positive temperatures the NO content almost did not change. At the same time, their resistance to negative temperatures did not develop after cold exposure to 4°C, which indicates the role of endogenous nitric oxide in plant adaptation to cold (Zhao et al., 2009).

A significant increase in nitrate reductase activity and transcripts of its genes was shown in Arabidopsis and citruses exposed to low positive temperatures (Puyaubert and Baudouin, 2014). The effect of increasing the content of nitric oxide in Arabidopsis was leveled by the action of the nitrate reductase inhibitor tungstate, but not by treatment with an inhibitor of enzymes of the oxidative pathway of NO synthesis L-NAME (N^G^-nitro-L-arginine methyl ester) (Cantrel et al., 2011). On the other hand, in pea (*Pisum sativum*), cold-induced NO formation was suppressed by L-NAME (Puyaubert and Baudouin, 2014). Therefore, in general, cold stress-induced nitric oxide generation in plants, apparently, can be carried out by different enzyme systems and have specific features (Yemets et al., 2019).

### Nitric oxide synthesis in plants exposed to high temperatures

In response to high temperatures, an increase in nitric oxide content in plants of different species has also been recorded. For example, even at moderately raised temperatures (+30°C), pea plants showed an increase in nitric oxide in tissues (Corpas et al., 2008). In rice (*Oryza sativa*) leaves, increased nitric oxide content was recorded after two hours of exposure to +38°C (Song et al., 2013). When exposed to +42°C, an increase in NO amount was found in *Citrus aurantium* (Ziogas et al., 2013) and strawberry (*Fragaria* × *ananassa*) (Christou et al., 2014). After a 1-minute hardening heating of wheat (*Triticum aestivum*) seedlings at +42°C, a rise in nitric oxide content was noted in the roots with a maximum after 30-60 min (Karpets et al., 2015a). This effect was almost completely eliminated by animal NOS inhibitor L-NAME and partially by nitrate reductase inhibitor sodium tungstate, indicating the likely involvement of both the oxidative and reductive NO synthesis pathways under the action of high temperature.

Heat-induced NO fluorescence has been observed in cell of *Nicotiana tabacum*. The earliest increase in NO content was noted in plastids with a subsequent effect in the nucleus and cytosol (Gould et al., 2003). At the same time, some studies report a decrease in the amount of nitric oxide in plant cells some time after the action of stress factors (Parankusam et al., 2017). This may be due to the transient effect of increasing the NO content, which is characteristic of signaling processes.

The role of nitric oxide in the development of heat resistance in plants is indicated by the elimination of the effects of thermal hardening of wheat seedlings by the action of the NO scavenger PTIO (2-phenyl-4,4,5,5-tetramethylimidazoline-1-oxyl-3-oxide), as well as inhibitors of NO synthesis enzymes (Karpets et al., 2015b).

## Hydrogen sulfide synthesis in plants at extreme temperatures

### Enzymatic systems for synthesis of hydrogen sulfide

One of the main pathways for hydrogen sulfide synthesis in plants is the conversion of L-cysteine into pyruvate with the release of hydrogen sulfide and ammonium (Romero et al., 2013). This reaction is catalyzed by L-cysteine desulfhydrase (L-CD). This enzyme has been detected in plants of various taxonomic groups (Zhang et al., 2021). An enzyme with this activity is localised in the cytoplasm, plastids, and mitochondria (Li, 2013). Proteins with chloroplast and mitochondrial localisation are regarded as a special kind of L-CDes – NifS-like proteins (Zhang et al., 2021). Hydrogen sulfide can also be formed from D-cysteine by D-cysteine desulfhydrase in the cytoplasm (Guo et al., 2016).

It is known that H_2_S can be a by-product of cysteine biosynthesis and an intermediate product in sulfate assimilation (Birke et al., 2015). The process of cysteine biosynthesis involves two steps. First, serine acetyltransferase (SAT) catalyzes the biosynthesis of the intermediate O-acetylserine (OAS) from acetyl-CoA and serine (Birke et al., 2012). Then, O-acetylserine (thiol) lyase (OASTL) enables sulfide incorporation into OAS to form cysteine (Álvarez et al., 2010). However, OASTL, which is a cysteine synthase (CS)-like protein, is involved predominantly in L-cysteine degradation rather than in its biosynthesis, because the K_m_ value associated with L-cysteine degradation is 13 times higher than that for L-cysteine biosynthesis (Álvarez et al., 2010). This protein is also found in the cytoplasm and is considered as another desulfhydrase, AtDES1 (Álvarez et al., 2010; Zhang et al., 2021).

It is also assumed that CBL protein is involved in hydrogen sulfide synthesis in plants (Wang et al., 2022b). The endogenous H_2_S content was shown to be lower in *Arabidopsis thaliana cbl* mutants than in the wild type.

Cyanoalanine synthase (CAS) is also considered to be one of the enzymes for H_2_S synthesis in plants. This mitochondria-localized enzyme generates cyanoalanine, produced alongside hydrogen sulfide, with cyanide and cysteine as substrates (Zhang et al., 2021). It is assumed that the main function of cyanoalanine synthase is associated with the control of toxic cyanide (Li, 2015).

In addition, H_2_S can be synthesized by sulfite reduction involving sulfite reductase (Li, 2013). This process requires reduced ferredoxin as a sulfur reducing agent. Finally, hydrogen sulfide can also be released during the decomposition of carbonyl sulfide by carbonic anhydrase contained in chloroplasts (Zhang, 2016).

In general, it is assumed that the relevance of different enzymatic properties depends on the specific subcellular localisation of hydrogen sulfide-producing enzymes (Zhang et al., 2021). The existence of complex and diverse mechanisms regulating H_2_S production and cysteine biosynthesis among plant species may in itself indicate the involvement of hydrogen sulfide in complex regulatory processes in plant cells (Li, 2020).

### Hydrogen sulfide synthesis in response to low and high temperatures

Several reports have shown that low temperatures increased expression of L/D-cysteine disulfhydrase genes and increased H_2_S content in leaves in Arabidopsis plants (Shi et al., 2015), increased synthesis of hydrogen sulfide in grapes (Fu et al., 2013) and raised H_2_S levels in Bermuda grass seedlings (Shi et al., 2013).

It was also reported that in *Lamiophlomis rotate*, a plant growing in cold mountain conditions, the expression of genes for enzymes involved in H_2_S biosynthesis (*OASTL*, *CAS*, *L/DCD*) increased with increasing altitude (4350, 4800, and 5200 m) (Ma et al., 2015a). The authors believe that this phenomenon indicates a role for H_2_S in cold tolerance of plants at high altitudes. The effect of increased hydrogen sulfide content has also been found in some warm weather species. Thus, H_2_S synthesis was increased in cucumber leaves in response to 4°C (Liu et al., 2019). Also, on example of cucumber plants, it was shown that endogenous and exogenous hydrogen sulfide induced a signal chain involving plant hormone indole-3-acetic acid (IAA) and hydrogen peroxide (Zhang et al., 2021). One function of this signal chain during cold stress may be to induce expression of transcription factor CBF1 gene and, consequently, cold-sensitive genes *COR47* (Zhang et al., 2021).

There is still little data on the involvement of endogenous hydrogen sulfide in plant adaptation to high temperatures. Prolonged exposure of plants to moderately high temperatures resulted in an increase in hydrogen sulfide content in the cells of strawberry (Christou et al., 2014), tobacco (*Nicotiana tabacum*) (Chen et al., 2016), and rice (Gautam et al., 2022). In roots of wheat seedlings, a transient increase in hydrogen sulfide content was noted after one-minute heating at a hardening temperature of 42°C (Havva et al., 2022). Such an effect was not observed when seedlings were treated with hydrogen sulfide scavenger hypotaurine or sodium pyruvate, a L-cysteine desulfhydrase inhibitor. At the same time, these H_2_S antagonists also eliminate the effect of increasing heat resistance of wheat seedlings.

## Post-translational modifications of proteins as the primary mechanism for the biological effects of NO, H_2_S and ROS

Following the biosynthesis in ribosomes, proteins can undergo numerous post-translational modifications (PTMs), which occur through the interaction of specific amino acid residues with certain compounds contained in the cellular environment (Corpas et al., 2022). The spectrum of PTMs is very diverse and includes phosphorylation, ubiquitination, methylation, glycosylation, acylation, alkylation, hydroxylation as well as specific reactions with protein thiol groups: S-sulfenylation, S-glutathionylation, S-nitrosation, persulfidation, S-cyanylation, and S-acylation. According to the UniProtKB/Swiss-Prot database, about 450 different PTMs have been identified.

PTMs can be either reversible or irreversible, in some cases they are carried out by specific modifying enzymes. Also, PTMs may occur spontaneously (non-enzymatic) depending on the physico-chemical properties of the reactive amino acids and characteristics of the cellular environment (e.g. pH, metabolites, etc.) (Valderrama et al., 2019). Notably, Cys, Lys, and N-terminal residues are targets for several PTMs (Friso and van Wijk, 2015).

Among the compounds that interact with proteins, gasotransmitters and ROS play a special role. PTMs serve as a mechanism for regulating functional activity of proteins due to the direct influence of signaling molecules on them, as well as the main mechanism for realizing signaling potential of gasotransmitters and ROS.

### ROS-induced protein PTMs

ROS means a set of mutually convertible reactive oxygen species, most of which exist for a short time. These include free radical particles – superoxide anion radical ( ${\text{O}}_{2}^{\cdot –}$ ), hydroxyl radical (ON**^•^**), peroxide radicals ( ${\text{RO}}_{2}^{\cdot –}$ , etc), and neutral molecules such as hydrogen peroxide (H_2_O_2_), singlet oxygen (^1^O_2_), etc (Mittler et al., 2022). Among ROS, hydrogen peroxide has the highest signaling potential. It is believed to play a crucial role in oxidative signaling (Mittler et al., 2022). The main participants in ${\text{О}}_{2}^{\cdot –}$ /H_2_O_2_ generation in plants are photosynthetic electron transport chain (chloroplasts), photorespiration process (peroxisomes), respiratory electron transport chain in mitochondria, and plasmalemma-localized NADPH oxidase (Gautam et al., 2017).

ROS-induced PTMs are usually more common in those compartments where ROS are produced. Cys-oxidation processes play a vital role in redox homeostasis of plant cells. The initial oxidation of Cys with hydrogen peroxide leads to the formation of sulfenic acid (R-SOH), which can later be oxidized to sulfinic (R-SO_2_H) and sulfonic (R-SO_3_H) acids (Valderrama et al., 2019). The oxidation of thiols to sulfinic acid is believed to be largely irreversible; however, these groups can be reduced by the ATP-dependent enzyme sulfiredoxin (Rey et al., 2007). Oxidation to sulfonic acid is definitely irreversible.

S-sulfenylation of Cys by hydrogen peroxide is primarily regarded as a mechanism for regulating functional activity of proteins. Under *in vitro* conditions, S-sulfenylation of about 800 polypeptides, including such important regulatory proteins as protein kinases, phosphatases, acetyltransferases, deacetylases, and deubiquitinases, has been demonstrated using human colon carcinoma cell line (Yang et al., 2014). In plants, 1394 potentially S-sulfenylation-sensitive targets were detected using a special highly reactive BTD probe (1-(pent-4-yn-1-yl)-1 Hbenzo[c][1,2]thiazin-4(3H)-one 2,2-dioxide) (Huang et al., 2019).

Despite the considerable progress in understanding the mechanism of post-translational modification of proteins under the ROS action, the contribution of this mechanism to the regulation of the state of specific proteins important for the adaptation of plants to stressful temperatures has not yet been sufficiently studied. It is a fact that in prokaryotes, H_2_O_2_ can oxidize thiol groups directly in transcription factor proteins (for example, Oxy R) (Vranova et al., 2002). In eukaryotes, the regulation mechanism of transcriptional activity involving ROS is more complex and includes a complex of proteins and peptides (Ndamukong et al., 2007; Lushchak, 2011).

However, experimental data accumulated over the last decade indicate the possibility of direct ROS-mediated activation of HSF (heat shock factors) under heat stress not only in prokaryotes but also in higher plants (Haider et al., 2021). For example, the transcription factor HSFA2 has been shown to be strongly induced at the transcriptional level in response to heat stress and H_2_O_2_ treatment (Nishizawa et al., 2006). It was also found that HSFA1a was activated by trimerization not only in response to heat stress, but also to the H_2_O_2_ action both *in vivo* and *in vitro* (Liu et al., 2013a). Heat stress-induced expression of HSP17.7 and HSP21 was found to be impaired in RBOH (respiratory burst oxidase homolog –catalytic subunit of NADPH oxidase) mutants (*rbohB*, *rbohD*, and *rbohB/D*), indicating a heat stress-induced HSF-ROS interaction (Wang et al., 2014). However, more research is still required to directly identify which amino acid residues of HSF interact with ROS, leading to their activation.

A specific example of the regulatory role of hydrogen peroxide-induced PTM is the oxidation of Cys in transcription factor protein (ethylene-sensitive group VII factor, ERFVII), which plays a key role in altering gene expression under hypoxia and probably heat stress. In the presence of oxygen, cysteine residues of ERFVII are oxidized to sulfenic acid, conjugated with arginine, and sent to proteasomes for degradation. However, under conditions of low oxygen content, ERFVII is released from the plasma membrane and moves to the nucleus, where it activates the expression of hypoxia response genes (Dietz, 2014). On the other hand, such a PTM may be a positive regulator of gene transcription, promoting the translocation of heat shock protein transcription factors (HSF) from the cytosol to the nucleus following the oxidation of Cys by hydrogen peroxide (Habibi, 2014).

A review by Sun and Guo (2016) provides many examples of the induction of various HSPs in chloroplasts and mitochondria under the influence of H_2_O_2_. Using genome-wide analysis of the Arabidopsis catalase-deficient mutant, a number of genes encoding transcription factors regulating synthesis of specific small HSP were found to be activated by hydrogen peroxide (Vandenabeele et al., 2004).

The influence of ROS on the state of transcription factors can also be realized indirectly, primarily through the processes of phosphorylation/dephosphorylation. The effect of ROS oxidation of cysteine residues in protein kinases and protein phosphatases is well known (Gupta and Luan, 2003). The participation of H_2_O_2_ in the control of tyrosine phosphorylation of plant proteins has also been shown (Karimova and Petrova, 2007). At the same time, according to the authors, endogenous hydrogen peroxide affects both the activity of tyrosine protein phosphatases (inhibits them by oxidising the SH-groups of the catalytic centre) and the activity of tyrosine protein kinases (oxidation of sulfhydryl groups activates these enzymes).

### NO-induced protein PTMs

**Figure 1:**
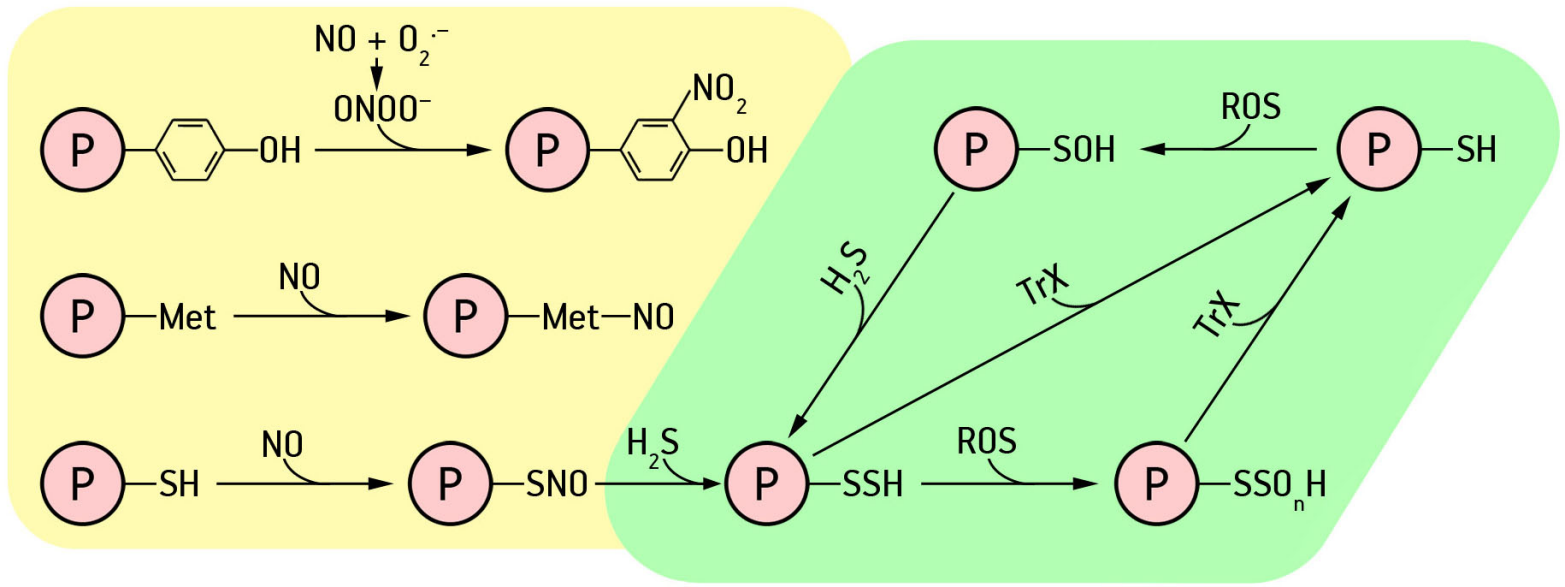
Post-translational modifications of proteins by the action of hydrogen sulfide and nitric oxide. Explanations in the text.

Nitric oxide can act directly or *via* derivative molecules (reactive nitrogen species, RNS) to induce various PTMs, including tyrosine (Tyr) nitration, metal nitrosylation, and S-nitrosation of Cys (Mishra et al., 2021) (**Figure 1**).

Protein tyrosine nitration is a selective process consisting of the accession of a nitro group (-NO_2_) to one of the two equivalent *ortho*-carbons in the aromatic ring of tyrosine residues to form 3-nitrotyrosine (Valderrama et al., 2019). Tyrosine nitration has traditionally been considered an irreversible mechanism and a marker of nitrosative stress (Corpas and Barroso, 2013). However, the existence of tyrosine denitrase, which reduces 3-nitrotyrosine in mammalian cells, points to a role for tyrosine nitration in NO-mediated signaling in these cells. Yet, the specific denitrase protein has not been identified in plants and there is currently no information on these issues (Valderrama et al., 2019).

Nevertheless, in recent years, it has been possible to show that protein nitration can be involved in regulatory processes. Analysis of the nitration of different organs of pea (*Pisum sativum* L.) plants during development and senescence indicates a specific protein nitration pattern for each organ (Begara-Morales et al., 2013). In addition, site-directed mutagenesis confirmed that Tyr-198 of NPR1 from Arabidopsis is the major nitration site responsible for the inhibition of its enzymatic activity by peroxynitrite (ONOO^.-^). Based on these results, the authors suggested that peroxisomal NO metabolism may contribute to the regulation of physiological processes in the absence of stress (Mata-Pérez et al., 2016a; Mata-Pérez et al., 2016b). The effect of tyrosine nitration in molecules of α-tubulin, the main protein of cytoskeleton components in plant cells, was also found, which may be associated with the regulation of dynamic properties of microtubules, directly involved in growth and division of plant cells (Blume et al., 2013).

S-nitrosation is the covalent attachment of the NO fragment to the reactive cysteine (Cys) of a protein to form S-nitrosothiol (SNO) (**Figure 1**). S-nitrosation is a reversible process considered to be a cellular switch that regulates functions of target proteins (Mishra et al., 2021). Since the SNO bond is redox sensitive, it can be cleaved by intracellular reducing agents such as glutathione and ascorbate, as well as reduced metal ions (Zhang and Liao, 2019). Moreover, S-nitrosation of target cysteine residues promotes the formation and breaking of intermolecular disulfide bridges, causing conformational changes in target proteins, and also affects cofactor binding (Astier et al., 2012). This type of PTM can also affect protein activity, stability, subcellular localization, and protein-protein interactions necessary for the regulation of physiological functions under certain conditions (Zhang and Liao, 2019).

In Arabidopsis plants, using a site-specific nitrosoproteomic approach, 926 proteins were found that can be targets for S-nitrosation (Hu et al., 2015). The effect of S-nitrosation of 44 proteins was detected in Arabidopsis under cold stress (Puyaubert et al., 2014). However, in general, data on S-nitrosation of specific proteins involved in adaptation to stress temperatures are still insufficient.

Another NO-induced PTM is metal nitrosylation. This effect consists of an interaction of NO with transition metals such as iron or copper present in metalloproteins, including phytohemoglobin, catalase, or cytochrome oxidase (Mishra et al., 2021). Formation of metal nitrosyl complexes causes reversible conformational changes in proteins and changes their structure and/or functional activity (Arora et al., 2016). However, little is known about the significance of this PTM in higher plants.

One of the best studied enzymatic proteins whose activity is regulated by nitric oxide post-translational modifications is cytosolic ascorbate peroxidase (cAPX) (Correa-Aragunde et al., 2015). Thus, S-nitrosylation of Cys-32 was found to stimulate cAPX activity. Cys-32 is present in 100% of cAPX described so far and is part of the pocket that binds ascorbate (Correa-Aragunde et al., 2013). At the same time, the reversible binding of NO to the heme prosthetic group is known to inhibit cAPX (Clark et al., 2000). Another potential redox modification of cAPX is the nitration of tyrosine residues (Lozano-Juste et al., 2011). This modification occurs in Tyr-5 and Tyr-235 of cAPX, causing irreversible inhibition of enzyme activity (Begara-Morales et al., 2013). In addition to the action of these modifications separately, simultaneous S-nitrosylation, tyrosine nitration, and carbonylation of cAPX are also possible. This phenomenon can occur under the influence of severe oxidative stress, and it leads to the degradation of the enzyme molecules by ubiquitin (Correa-Aragunde et al., 2015). This is precisely the phenomenon recorded in the heat shock effect on Bright Yellow-2 tobacco cells (de Pinto et al., 2013).

Catalase has been studied in considerable detail as a possible target of modifications by NO. It was found that NO can bind to iron in the heme, which prevents the binding of hydrogen peroxide from to the metal ion, thereby inhibiting catalase activity (Arora et al., 2016). On the other hand, the activation of catalase by nitric oxide through the S-nitrosylation process has also been reported (Bai et al., 2011).

There is also evidence that nitric oxide can inhibit NADPH-oxidase through S-nitrosylation of cysteine (Cys-890) (Yun et al., 2011). This may be another mechanism of NO involvement in regulating cell redox homeostasis.

Nitric oxide PTMs of NADPH-generating enzyme molecules have been reported in recent years (Corpas et al., 2021), in particular, of ferredoxin-NADP reductase (FNR). This enzyme is considered one of the main sources of NADPH in chloroplasts. Various proteomic analyses identified FNR as a target for Tyr nitration and S-nitrosation.

Increased FNR protein synthesis in sunflower seedlings under high-temperature stress was shown. However, FNR activity was wherein reduced, and *in vitro* analysis of FNR activity in the presence of ONOO-donor SIN-1 showed inhibition of enzyme activity (Chaki et al., 2011). In this work it was found that sunflowers were subjected to nitro-oxidative stress at high temperatures, resulting in impaired NADPH generation. Thus, tyrosine nitration inhibits FNR (Chaki et al., 2011). At the same time, it remains unclear how FNR activity will change due to the S-nitrosation of cysteine residues (Niu et al., 2019).

NADP-glyceraldehyde-3-phosphate dehydrogenase (NADP-GAPDH) is another enzyme involved in the formation of NADPH, various forms of which are localized in the cytoplasm and chloroplasts (Corpas et al., 2021). Both tyrosine nitration and S-nitrosation of the enzyme lead to the inhibition of its activity. The S-nitrosation at Cys-149 was found to be reversible; the reverse reaction is mediated by GSH (Zaffagnini et al., 2013).

Another mechanism of NADPH synthesis is related to the activity of enzymes in the oxidative phase of the pentose phosphate pathway, primarily glucose-6-phosphate dehydrogenase (G6PDH). Using nitric oxide donors and proteomics techniques, G6PDH inhibition of pea (*Pisum sativum*) leaves was shown to be possible both through nitration of tyrosine residues and through S-nitrosation of cysteine (Corpas et al., 2021). However, *in vivo* experiments have shown increased activity and expression of G6PDH genes in soybean (*Glycine max*) roots under drought conditions when treated with NO donor (Wang et al., 2020).

There is also evidence of nitric oxide donors reducing the activity of several other enzymes involved in NADPH synthesis. In particular, a decrease in the activity of the NADP-malic enzyme during Tyr-73 nitration has been shown (Begara-Morales et al., 2019). Moreover, this effect is considered a component of the development of nitrosative damage in *A. thaliana* under cold stress. A decrease in NADP-isocitrate dehydrogenase activity has also been shown in the presence of NO donors causing the effects of tyrosine nitration and S-nitrosation of cysteine residues (Corpas et al., 2021). Thus, *in vitro* NO modifications of the enzyme molecules involved in NADPH generation mainly cause their inhibition. However, *in vivo* it is possible for nitric oxide to activate the gene expression of individual enzymes involved in maintaining the NADPH pool.

### H_2_S-induced protein PTMs

It is currently believed that the signaling effects of hydrogen sulfide, which constitute many physiological and pathological processes in mammals and plants, are related to protein persulfidation – conversion of cysteine thiol group (-SH) into corresponding persulfide (-SSH) (Yuan et al., 2017; Filipovic et al., 2018; Paul and Snyder, 2018). However, the mechanism for this process is still debated. It is assumed that H_2_S or its ionic forms HS^−^ and S^2−^ cannot react directly with protein thiols. This interaction requires the presence of oxidizing agents (Aroca et al., 2021). It is more likely that H_2_S can react directly with oxidised cysteine residues (R-SOH). Protein nitrosothiols (RSNO) SNO groups can also react with H_2_S to form protein persulfides (Paul and Snyder, 2012; Xuan et al., 2020). However, there is evidence that this process is thermodynamically unfavourable (Filipovic et al., 2012). Therefore, reaction of H_2_S with sulfenic acid residues to form protein persulfides is considered the most likely effect of H_2_S.

Oxidation of cysteine residues is a method of redox control of proteins functional activity (Cuevasanta et al., 2015). Thus, it is assumed that the process of protein modification is triggered by ROS signal, i.e., oxidation of thiol group of cysteine to sulfenic acid with H_2_O_2_ (Aroca et al., 2021). Sulfenic residues are easily persulfided with hydrogen sulfide to form persulfide groups (R-SSH). Protein sulfenic residues have been shown to react two orders of magnitude faster with H_2_S than with glutathione (Cuevasanta et al., 2015). Persulfidation is also a mechanism to protect proteins from oxidative damage. Persulfided residues involving the thioredoxin system (Trx/TrxR) can be converted into conventional sulfhydryl groups (Filipovic et al., 2018). Schematically, the processes of formation of sulfene and persulfide groups, as well as reduction of thiol groups, can be depicted as follows:


(1) protein−SH+ROS→protein−SOH

(2) $$\text{protein}-\text{SOH}+{\text{H}}_{2}\text{S}\to \text{protein}-\text{SSH}$$

(3) protein−SSH+Trx/TrxR→protein−SH

In the proteome of *A. thaliana* treated with NaHS, 106 persulfided proteins have been identified that are mainly involved in photosynthesis, protein synthesis, cellular organization, and a primary metabolism (Aroca et al., 2018). Using another technique of proteomic analysis of endogenous persulfated proteins in *A. thaliana* wild-type and *des1*-mutant leaves, 2015 persulfated proteins were identified, which were mainly involved in the regulation of the primary metabolism, abiotic and biotic stress responses, plant growth, and development processes (Aroca et al., 2017).

The possibility of regulating through the persulfidation process the activity of several enzymes involved in maintaining the pool of a key cellular reducing agent, NADPH, is being considered (Corpas et al., 2021). In particular, NADP-GAPDH activity was found to be increased by H_2_S (Aroca et al., 2015). On the other hand, inhibition of NADP-isocitrate dehydrogenase and NADP-malic enzyme due to persulfidation has been shown (Muñoz-Vargas et al., 2018; Muñoz-Vargas et al., 2020).

Persulfidation is probably a part of the toolkit for regulation of gene expression. A transcriptomic study carried out on Arabidopsis plants showed that treatment with exogenous H_2_S resulted in significant changes in expression of numerous genes. When plants were treated with hydrogen sulfide, in particular, the expression of genes encoding various regulatory transcription factors was enhanced (Sen et al., 2012; Aroca et al., 2017). A study of tomato gene expression during NaHS root treatment showed that 5349 genes were activated and 5536 ones inhibited (Guo et al., 2018). A number of studies have also shown a role for sulfide in modifying histones and altering chromatin structure that constitutes epigenetic regulation (Shivaraj et al., 2020).

## Influence of gasotransmitters on functioning of plant protective systems at extreme temperatures

### Modification of stress-protective systems with nitric oxide

As already mentioned, cold stress induces increased nitric oxide synthesis in plants. This effect appears in most cases to be one of the signals needed to trigger adaptive responses. Thus, the expression of specific cold-sensitive genes such as *CBF1*, *CBF2*, *CBF3*, *LTI30*, *LTI78*, *COR15a* has been shown to be NO-status dependent (Puyaubert et al., 2014; Baudouin and Jeandroz, 2015). The cold-induced enhancement of expression of these genes was suppressed by NO scavenger PTIO and was weakly manifested in mutants defective in nitrate reductase genes. During cold acclimation of tomato, expression of the protein kinase CPK27 gene turned out to be dependent on NO (Lv et al., 2018).

Zhang et al. (2019) investigated the role of NO signals in cold acclimatization of two alfalfa species, *Medicago falcata* (resistant) and less resistant *Medicago truncatula*. The cold acclimatization effect of both species was suppressed when the plants were treated with nitrate reductase inhibitor tungstate and the NO scavenger PTIO. Cold exposure increased the number of NIA1 NR isoform transcripts, but not NIA2. However, in the more resistant species *M. falcata*, this effect was more pronounced. Treatment with NO donors, as well as cold acclimatisation, led to an increase in cold resistance of both species. Wherein the expression of key antioxidant enzyme genes, *Cu/Zn-SOD2*, *Cu/Zn-SOD3*, catalase, and chloroplast ascorbate peroxidase (*APX1*), was induced under the influence of cold and NO donor. These effects were more pronounced in *M. falcata* than in *M. truncatula*. Thus, the authors have shown an association between NO generation, antioxidant system performance, and cold tolerance of alfalfa species (Zhang et al., 2019).

The positive effect of NO on plant cold resistance may be due to S-nitrosation of target proteins, including antioxidant enzymes. Thus, in *Brassica juncea*, Fe-SOD was identified as a target for S-nitrosation under cold stress conditions (Sehrawat et al., 2013). Overall, *B. juncea* showed the effect of differential S-nitrosation of 10 proteins, among which, apart from SOD, also dehydroascorbate reductase and glutathione-S-transferase (Sehrawat and Deswal, 2014). The possibility of increasing the activity of catalase and non-specific peroxidases due to their post-translational modifications by NO under cold stress and the association of these effects with resistance of plants to hypothermia has also been reported (Sougrakpam et al., 2018).

Processes associated with S-nitrosation of antioxidant enzymes also occur in the apoplast. It is assumed that the PTM of apoplastic ascorbate peroxidase, which leads to an increase in its activity, may be associated with the further development of cold resistance in plants (Sehrawat and Deswal, 2014). Importantly, NO not only interacts directly with protein molecules of antioxidant enzymes, but is also involved in enhancing gene expression of these proteins during cold adaptation (Puyaubert et al., 2014). This allows us to consider NO as one of the key signaling mediators involved in the maintenance of redox homeostasis under cold stress.

Treatment of Bermuda grass (*Cynodon dactylon*) plants with the NO donor sodium nitroprusside (SNP) reduced the electrolyte release from tissues caused by cold and prevented an increase in the lipid peroxidation product malondialdehyde content in cells. At the same time, the NO-donor-treated plants showed higher values of SOD, peroxidase, and catalase activity at low temperatures (Fan et al., 2015). Priming of seeds of winter wheat and rye with SNP contributed to an increase in activity of SOD and guaiacol peroxidase (Kolupaev et al., 2020). Protective effects of the nitric oxide donor were clearly evident in seedlings subjected to cold hardening. After cold hardening and especially after freezing, the content of malondialdehyde (lipid peroxidation product) in seedlings grown from seeds primed with SNP was lower compared to the corresponding controls. Consequently, there is reason to believe that nitric oxide enhances cold-induced activation of cereal antioxidant system (Kolupaev et al., 2020).

It is noteworthy that NO can affect many components of antioxidant defense. Thus, exposure to NO mitigated the negative effects of cold stress on peach fruit by stimulating alternative oxidase and reducing oxidative damage (Song et al., 2021).

Cold-induced Δ^1^-pyrroline-5-carboxylate synthase gene expression and proline accumulation in *A. thaliana* were also found to be mediated by NO (Zhao et al., 2009). These effects were weakly expressed in the *nia1nia2* mutants and were suppressed by the NO scavenger PTIO.

As mentioned above, the adaptation of plants to the stress effect of high temperatures, similarly to adaptation to low temperatures, includes the activation of gene expression and increased activity of antioxidant enzymes as well as accumulation of low-molecular-weight antioxidants and multifunctional compounds that also exhibit antioxidant properties. Nitric oxide can enhance such effects. The example of wheat plants shows an increase in the activity of SOD, catalase, guaiacol peroxidase, ascorbate peroxidase, glutathione reductase under heat stress as a result of pretreatment of plant objects with NO donor SNP (Karpets et al., 2011; El-Beltagi et al., 2016). Under prolonged heat stress, SOD, catalase, ascorbate peroxidase, and glutathione reductase activities increased in wheat treated with SNP (Iqbal et al., 2022). The activities of SOD, ascorbate peroxidase, and glutathione reductase increased in callus tissues of wheat under the influence of SNP in addition to the increase in these indicators caused by heat stress (El-Beltagi et al., 2016). The increase in heat resistance of *Phaseolus radiatus* and *Phragmites communis* under the action of NO donors was also accompanied by an increase in activity of antioxidant enzymes (Yang et al., 2006; Song et al., 2008). Treatment of strawberry plants with a nitric oxide donor increased their heat resistance, which was manifested in a decrease in H_2_O_2_ accumulation, a decrease in lipid peroxidation under stress conditions, and an increase in activity of enzymatic antioxidants (Manafi et al., 2021).

In *Zea mays* plants, an increase in ascorbate peroxidase activity under normal conditions and under heat stress has been shown by treatment with the NO donor SNP (Sun et al., 2022). However, the nitric oxide donor did not affect *APX1* gene expression. At the same time, both an increase in glutathione reductase activity and an increase in *GR1* gene expression under the influence of exogenous NO were shown.

An increase in the content of multifunctional protective compounds that also have antioxidant activity – proline and trehalose – was found under the influence of SNP treatment in wheat plants exposed to long-term heat stress (Ju and Ma, 2011; Han et al., 2013; Iqbal et al., 2022). Spraying rice plants with SNP mitigated the manifestation of oxidative stress under high temperature conditions by increasing the content of ascorbate, reduced glutathione, and increasing the accumulation of proline (Gautam et al., 2021). Alamri et al. (2019) revealed an endogenous NO-dependent increase in proline content in horse beans (*Vicia faba*) in response to high temperature. This effect was inhibited when plants were treated with NO scavenger cPTIO (Alamri et al., 2019).

In general, a fairly large pool of results has been accumulated in the literature, indicating an increase in the enzymatic antioxidant system of plants and the accumulation of low-molecular antioxidants under the action of NO. Such effects may be due to both post-translational modifications and the effect of nitric oxide on the expression of genes for antioxidant enzymes and enzymes involved in synthesis of low-molecular-weight protective compounds.

However, there are also a large number of contrary phenomena, without understanding the reasons for which it is impossible to build a complete picture of NO participation in the regulation of the antioxidant system. For example, the NO donor SNP has been shown to reduce ascorbate peroxidase and glutathione reductase activity in cotton callus (Vital et al., 2008). The possible mechanisms of this phenomenon were not explained in the above work, but it is possible that that effect took place due to the predominance of those post-translational modifications by NO that inhibit these enzymes. On the other hand, along with numerous reports on a decrease in the activity of antioxidant enzymes when plants were treated with NO antagonists, some studies have revealed an increase in the activity of antioxidant enzymes under the influence of nitric oxide scavengers. Using roots of intact wheat seedlings, treatment with NO donor, as well as a one-minute hardening at 42°C, was found to cause an increase in SOD, catalase, and guaiacol peroxidase activities (Karpets et al., 2015b). At the same time, treatment of such seedlings with NO scavenger PTIO and L-NAME, an inhibitor of nitric oxide synthesis along the oxidative pathway, also increased the activity of these enzymes, although it did not affect the increasing activity of these enzymes under the influence of hardening temperature (Karpets et al., 2015b). Thus, the opposite effects – a decrease in nitric oxide content when plants are treated with PTIO or L-NAME and an increase in nitric oxide (when plants are exposed to hardening heat or NO donor treatments) – can cause a phenomenologically similar effect: an increase in activity of antioxidant enzymes. Since different post-translational modifications of antioxidant enzyme molecules by nitric oxide can lead to different results (increased or decreased activity, see above), it is likely that, depending on the experimental conditions, both increased and decreased antioxidant enzyme activities are possible under the influence of NO donors.

Unusual aspects of nitric oxide regulation of protective systems were found in studies using NO-deficient *nia1,2noa1-2* mutants of Arabidopsis (Costa-Broseta et al., 2018). Unlike wild-type plants, these mutants exhibited constitutive frost resistance and were characterised by increased levels of low-molecular-weight antioxidants – ascorbate, reduced glutathione, flavonoids, and sugars. The authors concluded that NO action can attenuate synthesis and accumulation of antioxidants, osmoprotective and regulatory metabolites. It is noteworthy that rice mutants with a reduced activity of one of nitrate reductase forms (*nia2*) turned out to be more drought-resistant, with higher expression of the *APX2* and *CATA* genes of antioxidant enzymes (Zhou et al., 2021). Based on these data, the authors concluded that the *NIA2* gene negatively regulates drought tolerance in rice. However, it should be noted, that the *NIA2* form of nitrate reductase is not considered the main producer of NO and its content was not monitored in this work, making it impossible to unambiguously associate the observed phenomena with NO deficiency.

On the other hand, *hot5* mutants of Arabidopsis, defective in GSNOR activity, are known to be extremely sensitive to high temperatures (Lee et al., 2008). This effect has been attributed to an excess of nitric oxide in their cells when exposed to high temperatures. Notably, the sensitivity of *hot5* mutants to high temperatures was partially mitigated by the NO scavenger cPTIO treatment.

Despite the existence of such examples of negative regulation of plant adaptive responses by nitric oxide, in general, the literature accumulates significantly more examples of positive regulation of plant stress-protective systems by the action of NO. One mechanism of their activation may be related to the effect of nitric oxide on the expression of genes from the families of transcription factors controlling the complex defense response. Thus, when rice was treated with the nitric oxide donor SNP, an increase in the expression of genes of several transcription factors of the MYB and WRKY families was detected, which provide resistance to various stresses, including dehydration and extreme temperatures (Singh et al., 2017).

In general, it is likely that modifications in the NO status of plants, or even more broadly in nitrogen metabolism, can lead to significant changes in functioning of protective systems, including antioxidant ones (Hancock and Veal, 2021). Apparently, its activation can occur either through the direct involvement of NO or through the action of other signaling mediators, including calcium and hydrogen sulfide. H_2_S can function both synergistically and antagonistically with NO (see below). It is possible that, under certain conditions, a deficiency of NO or other nitrogen compounds may be a signal to activate other mechanisms that effectively regulate the functioning of the protective systems.

### Modifications of stress-protective systems with hydrogen sulfide

As noted earlier, the plant response to low temperatures is usually accompanied by an increase in gene expression and an increase in L/D-cysteine disulfhydrase activity and, consequently, endogenous hydrogen sulfide content. Using Arabidopsis mutants defective in L/D-cysteine desulfhydrase genes, the role of hydrogen sulfide in the activation of cold-sensitive *COR15* and *CBF3* genes has been shown (Du et al., 2017). One of the mechanisms of hydrogen sulfide involvement in the Arabidopsis cold stress response involves persulfidation of at least one component of the MAP-kinase signaling cascade (MAP4), resulting in the increased activity of this enzyme and activation of related signaling processes (Du et al., 2017).

Evidence for the stimulation by hydrogen sulfide of protective systems important for providing plant cold resistance is obtained mainly by the application of exogenous hydrogen sulfide donors (mainly NaHS). For example, NaHS treatment of *C. dactylon* increased plant survival after exposure to sub-zero temperatures, which was accompanied by the activation of antioxidant enzymes, such as SOD, catalase, guaiacol peroxidase, and glutathione reductase, and the accumulation of low-molecular-weight antioxidants of ascorbate-glutathione cycle, and osmolytes (Shi et al., 2013).

An increase in survival after freezing of winter wheat seedlings was observed when they were pretreated with 0.1 or 0.5 mM NaHS. One of the components of stress-protective effect of H_2_S donor was the phenylalanine ammonia lyase-dependent accumulation of flavonoid compounds with high antioxidant activity and reduction of the effects of oxidative stress (Kolupaev et al., 2018; Kolupaev et al., 2019a). Grapes (*Vitis vinifera*) under the influence of exogenous hydrogen sulfide at low temperatures showed an increase in SOD activity (Fu et al., 2013). In Arabidopsis plants, treatment with H_2_S donor under stressful conditions reduced not only ROS but also the content of active forms of nitrogen by increasing the activity of S-nitrosoglutathione reductase (GSNOR) (Shi et al., 2015).

A number of studies have shown an increase in resistance of fruits with hydrogen sulfide during low-temperature storage. Thus, cucumber fruits treated with hydrogen sulfide exhibited lower ROS levels and higher antioxidant enzyme activity during low-temperature storage (Wang et al., 2022a). Also, hydrogen sulfide treatment increased activity of main enzymes involved in energy metabolism, including cytochrome *c* oxidase, succinate dehydrogenase, H^+^-ATPase, and Ca^2+^-ATPase. In addition, H_2_S was found to induce Δ^1^-pyrroline-5-carboxylate synthase activity and cause a decrease in proline dehydrogenase activity, which promotes proline accumulation. An increase in total content of phenolic compounds and the activity of phenylalanine ammonium lyase (was recorded in banana (*Musa*) fruits during low-temperature storage under the influence of the hydrogen sulfide donor NaHS (Luo et al., 2015). A total content of flavonoids and anthocyanins under the NaHS action was also increased during low-temperature storage of hawthorn (*Crataegus*) fruits (Aghdam et al., 2018).

As already mentioned, under the conditions of high as well as low temperatures, expression of genes and activity of enzymes involved in hydrogen sulfide synthesis was shown to increase, which, in turn, caused an increase in endogenous H_2_S content in plants of different species. Also, high temperatures cause an increase in gene expression and activity of GSNOR, which contributes to the removal of excess reactive nitrogen species and ROS, ultimately increasing the tolerance to a high temperature stress. Using poplar plants (*Populus trichocarpa*) as an example, it has been shown that inhibition of H_2_S biosynthesis reduces GSNOR activity with a consequent increase in oxidative damage in leaves induced by RNS and ROS (Cheng et al., 2018). These results suggest that one of the mechanisms of H_2_S effects on plant heat resistance may be related to the activation of GSNOR, which contributes to the reduction of oxidative stress induced by RNS and ROS.

There is also evidence that hydrogen sulfide enhances the complex of major antioxidant enzymes in plants under high-temperature stress. Thus, treatment of maize seedlings under normal cultivation conditions with hydrogen sulfide donor NaHS stimulated the activity of antioxidant enzymes (catalase, guaiacol peroxidase, SOD, and glutathione reductase) and increased antioxidants content (GSH and ascorbate) compared to control (Li et al., 2014). Under heat stress, all of the above physiological parameters decreased, but the treatment of corn seedlings with NaHS helped to maintain the activity of antioxidant enzymes and the content of low-molecular-weight antioxidants at a higher level. An increase in activity of Δ^1^-pyrroline-5-carboxylate synthetase and a decrease in activity of proline dehydrogenase with subsequent accumulation of proline were also shown on corn plants (Li et al., 2013a).

In heat-stressed strawberry plants, pretreatment with NaHS enhanced the expression of genes encoding enzymes for the synthesis of antioxidants (ascorbic acid and glutathione) and maintained a higher redox potential of ascorbate AsA and glutathione GSH (AsA/GSH). NaHS treatment of plants also increased gene transcription of antioxidant enzymes (ascorbate peroxidase, catalase, SOD, and glutathione reductase), as well as chaperones (HSP 70, HSP 80, and HSP 90) and aquaporins (Christou et al., 2014). It is noteworthy that treatment with exogenous hydrogen sulfide eliminated the heat stress-induced increase in NO content. Perhaps, this effect was aimed at preventing the development of nitrosative stress.

## Functional interaction of NO, H_2_S, ROS, and Ca^2+^ in the formation of plant adaptive responses to stress temperatures and other adverse factors

Signaling and regulatory effects of gasotransmitters and ROS are largely determined by their functional interaction with each other. These interactions can be divided into several levels: (a) ordinary chemical interaction of molecules with each other; (b) competition for common binding targets with biomacromolecules; (c) influencing on each other’s synthesis, which may involve many other signaling mediators (Kolupaev et al., 2019b).

Direct reactions between H_2_S and NO include formation of nitroxyl (HNO) and nitrosothiols (RSNO), which in turn can interact with biomacromolecules (Aroca et al., 2020). It has also recently been discovered that persulfides are able to produce NO using nitrite through intermediates such as polysulfide, SNO^–^ (thionitrite) and S_2_NO^–^ (perthionitrite, nitrosodisulfide) (Bailey et al., 2016; Marcolongo et al., 2016; Marcolongo et al., 2019). Consequently, the interaction of H_2_S and NO produces intermediates that may also be involved in cellular signaling (Aroca et al., 2020). However, the mechanisms of formation and, moreover, the biological activity of these compounds in plant cells is not yet fully understood.

Hydrogen sulfide also directly interacts with some ROS. For example, the removal of hydroxyl radicals in the reaction with hydrogen sulfide (HO^•^ + H_2_S/HS^−^ → S^•−^ + H_2_O), and the interaction of hydrogen peroxide with the hydrosulfide anion (H_2_O_2_ + HS^−^ → HSOH + OH^−^) are considered possible (Carballal et al., 2011). Such interactions can reduce the signaling potential of relevant molecules and thereby modulate cellular signals (Hancock, 2019). However, the real contribution of these processes to signal damping remains largely unexplored.

The second level of interaction between gasotransmitters and ROS is determined by the presence of common interaction sites with target proteins s. Most often, thiol groups play this role. Sun et al. (2020) proposed a model according to which an excess of nitric oxide in the cellular system creates conditions for the conversion of individual thiol groups of proteins into nitrosothiol ones. At high concentrations of hydrogen sulfide in cells, persulfidation of proteins is activated, and finally, with a certain balance of H_2_S and NO, the persulfidated groups can be converted into the original thiols using glutathione and ascorbate as reducing agents. As mentioned above, ROS are also involved in the interaction of hydrogen sulfide with thiol groups in proteins: oxidation of thiols by hydrogen peroxide to sulfenes promotes the interaction of molecules with H_2_S to form −SSH. It is assumed that the type of thiol group conversion and eventually protein activity will be determined by the probability of the above interactions and will depend on the local concentrations of H_2_S, NO, and ROS (Hancock, 2019). Not only enzymatic and signaling proteins, but also some transcription factors can undergo such ‘probabilistic’ modifications (Zhou et al., 2020). Specific examples of proteins modified by ROS, NO, and H_2_S, in particular antioxidant enzymes, are given above in the review.

Probably, the regulation of the activity of enzymes that are involved in the maintenance of the NADPH pool is also mediated *via* the crosstalk between NO and H_2_S. Thus, glyceraldehyde-3-phosphate dehydrogenase (NADP-GAPDH) is negatively regulated by tyrosine nitration and cysteine S-nitrosation, whereas cysteine persulfidation causes enzyme activation (Aroca et al., 2015; Corpas et al., 2021). Therefore, it is likely that local concentrations of NO and H_2_S may influence NADP-GAPDH activity and NADPH formation in specific cellular compartments.

The most difficult to study and interpret the results are the mechanisms of functional interaction of NO, H_2_S, and ROS related to their mutual influence on each other’s synthesis. Such effects involve both direct modification by active molecules of functional groups of enzymes that synthesize signal mediators, and the indirect influence of these molecules on the expression of genes encoding the corresponding enzymes. There seems to be a constant direct and reverse regulation mechanism at work in these processes. Thus, several channels of interaction between ROS and hydrogen sulfide as mediators are known. Hydrogen sulfide directly activates the key enzyme that generates the ROS signaling pool, NADPH oxidase, by persulfidation of two cysteine residues (Cys-825 and Cys-890) in the catalytic subunit (RBOH*D*) (Shen et al., 2020). On the other hand, Arabidopsis plants showed increased expression of L/D-cysteine desulfhydrase in response to H_2_O_2_ treatment (Shi et al., 2015). The *atrbohD*, *atrbohF*, *atrbohD/F* mutants, unlike wild-type plants, did not show increased formation of hydrogen sulfide in response to drought stress, indicating the role of ROS generated by NADPH oxidase in the activation of stress-induced H_2_S synthesis (Shi et al., 2015).

Very ambiguous functional relationships were also found between H_2_S and NO. There was evidence of reduced stress-induced NO accumulation in strawberry leaves under the influence of hydrogen sulfide (Christou et al., 2014). It is believed that this effect is due to the stimulating effect of H_2_S on the expression of the S-nitrosoglutathione reductase gene (Li, 2020). On the other hand, hydrogen sulfide has been shown to cause persulfidation of cysteine residues of nitrate reductase and reduce its activity (Zhou et al., 2021). Thus, on the one hand, the effects of antagonism between nitric oxide and hydrogen sulfide are possible, and on the other hand, NO may mediate the signal transduction induced by H_2_S. The involvement of ROS and NO as mediators in stress-protective action of the hydrogen sulfide donor is also indicated by the data obtained on wheat seedlings subjected to heating. Induction of wheat seedlings heat tolerance by the hydrogen sulfide donor NaHS was accompanied by a transient increase in hydrogen peroxide and nitric oxide content in the roots as well as by an increase in nitrate reductase activity (Karpets et al., 2020). The H_2_S donor-induced increase in NO content was not evident in the presence of the antioxidant dimethylthiourea and the NADPH oxidase inhibitor imidazole. At the same time, the increase in H_2_O_2_ in roots occurring during treatment with exogenous hydrogen sulfide was not eliminated by the NO scavenger PTIO and the nitrate reductase inhibitor tungstate (Karpets et al., 2020). It can therefore be assumed that H_2_O_2_ is above NO in the hydrogen sulfide signaling chain inducing the development of heat resistance.

However, there is also evidence that hydrogen sulfide mediates the effects of nitric oxide. For example, the treatment of maize seedlings with a nitric oxide donor, which induced the development of heat tolerance, was accompanied by increased expression of the gene encoding L-cysteine desulfhydrase (*LCD1*) and increased enzyme activity (Sun et al., 2022). Wherein, the hydrogen sulfide scavenger hypotaurine abolished the effect of the NO donor on L-cysteine desulfhydrase and the development of heat resistance in maize seedlings.

An ambiguous functional interaction between H_2_S and NO is also evident in their regulation of plant stomatal responses, important for adaptation to drought and stress temperatures. Thus, the stomata-closing effect caused by hydrogen sulfide can be mediated by nitric oxide. NO scavenger PTIO has been shown to eliminate stomatal closure induced by NaHS treatment in *Ipomoea batatas* plants (Hu et al., 2014). In Arabidopsis, the slow-acting hydrogen sulfide donor CYY4137 induced a stomatal aperture-reducing effect that was also reversed by NO antagonists (Honda et al., 2015). The assumption of a mediating role of nitric oxide in the stomatal effects of hydrogen sulfide is also consistent with the absence of sodium hydrosulfide effects on stomata of Arabidopsis mutants defective in activity of two forms of nitrate reductase (*nia1/nia2*), the main enzyme generating NO in plant cells (Scuffi et al., 2014). On the other hand, there is also evidence of reduced H_2_S accumulation in Arabidopsis epidermal cells after treatment with NO donors (Lisjak et al., 2010).

Overall, the available evidence suggests that the location of NO and H_2_S as mediators in signaling circuits may be different (Li et al., 2013b). Thus, the hydrogen sulfide scavenger hypotaurine abrogates the stress-protective effect of the NO donor SNP on rice plants under prolonged heat stress, indicating the H_2_S role as a mediator of exogenous NO physiological effects (Gautam et al., 2022). The possibility of a positive (synergistic) effect of NO and H_2_S on the resistance of wheat plants to long-term heat stress is evidenced by data on the strengthening of the positive effect of the donors of these gasotransmitters on the antioxidant system (activity of SOD, catalase, and ascorbate-glutathione cycle enzymes) when used together (Iqbal et al., 2021).

**Figure 2:**
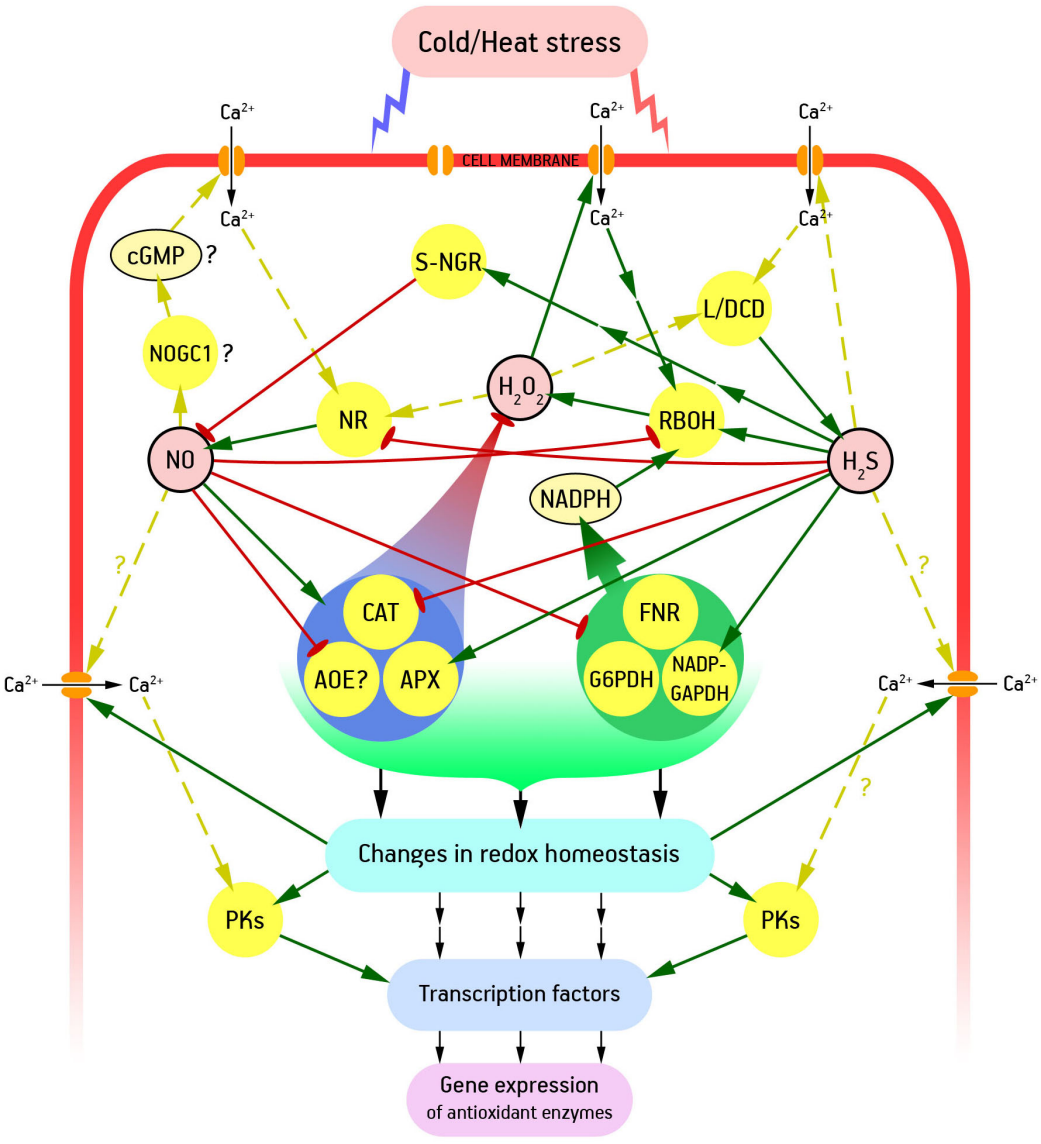
Pathways of functional interaction between H_2_S, NO, ROS and Ca^2+^ in formation of plant adaptive responses to low and high temperatures. AOE, antioxidant enzymes; APX, ascorbate peroxidase; CAT, catalase; cGMP, cyclic guanosine 3′-5′-monophosphate; FNR, ferredoxin-NADP reductase; G6PDH, glucose-6-phosphate dehydrogenase; L/DCD, L/D-cysteine desulfhydrase; NADP-GAPDH, NADP-dependent glyceraldehyde-3-phosphate dehydrogenase; NOGC1, NO-dependent guanilate cyclase 1; NR, nitrate reductase; PKs, protein kinases; RBOH, respiratory burst oxidase homolog (catalytic subunit of NADPH oxidase); S-NGR, S-nitrosoglutathione reductase. Interrupted arrows show indirect interactions between signalling participants, and dashed arrows show interactions whose mechanisms are poorly understood. Other explanations in the text.

Overall, ROS, nitric oxide, and hydrogen sulfide form a complex signaling network that ensures that appropriate adaptive responses are triggered. Another integral component of such a network is calcium as a universal intracellular messenger (Neill et al., 2008). As already noted, fluidization of membranes under heat stress and the opposite process of increasing their rigidity under cold stress led to a rapid entry of calcium into the cytosol. These changes in calcium homeostasis may be primary to changes in cellular ROS, NO, and H_2_S (Kolupaev et al., 2022) (**Figure 2**).

For instance, calcium ions are able to induce NO accumulation. The effects of plant nitrate reductase activation by calcium ions and inhibition in the presence of a calcium chelator *in vitro* have long been demonstrated (Sane et al., 1987). An increase in activity of this NO-synthesizing enzyme in intact plants under the influence of exogenous calcium has been also observed (Gao et al., 2011). Calcium entry into the cytosol can also be a stimulus for hydrogen sulfide synthesis. Thus, under the influence of exogenous calcium and calmodulin, activity of L-cysteine desulfhydrase was increased in cultured tobacco (*Nicotiana tabacum*) cells, which led to an increase in endogenous H_2_S formation (Li et al., 2015a). In addition, it is well known that calcium directly and indirectly activates NADPH oxidase, one of the main enzymes of ROS signaling (Baxter et al., 2014). Thus, temperature stress effects are likely to induce various components of the signaling network, including gasotransmitters and ROS (**Figure 2**).

These signaling mediators can promote the opening of calcium channels of different types. For example, potential-dependent calcium channels are known to be activated by ROS (Mori and Schroeder, 2004). Under the influence of NO, calcium channel proteins can undergo S-nitrosation, which also leads to increased calcium entry into the cytosol (Laxalt et al., 2007).

In addition to its possible direct effect on calcium channel status, nitric oxide may be involved in signaling chains as a secondary messenger functionally linked to calcium *via* cyclic guanosine 3′-5′-monophosphate (cGMP), cyclic adenosine diphosphate ribose (cADPR), and probably several other mediators (Singhal et al., 2021) (**Figure 2**). Despite the lack of clear molecular genetic evidence for the functioning of a cGMP-dependent signaling pathway in higher plants (Hancock and Veal, 2021), which connects NO and Ca^2+^ signaling in green algae (Singh et al., 2022), bioinformatics methods have provided evidence for the functioning of NO-dependent guanylate cyclase 1 (NOGC1) in *A. thaliana*. The recombinant NOGC1 protein has subsequently been shown to be able to synthesize cGMP in a NO-dependent manner, albeit in very small amounts (Gross and Durner, 2016). Overall, there is considerable experimental evidence indirectly suggesting the existence of NO-mediated cGMP formation in plant cells and the regulation of calcium homeostasis with its participation. A number of relevant examples are provided in recent reviews (Kolupaev et al., 2022; Praveen, 2022). It is *via* cGMP and calcium that many of the stress-protective effects of nitric oxide can be realized, in particular an increase in the heat resistance of plants (Karpets et al., 2016). A component of the latter is, in particular, a calcium-dependent increase in the activity of antioxidant enzymes (Karpets, 2017).

Calcium also appears to be involved in the transduction of H_2_S signals that induce the development of plant heat tolerance. The increase in heat resistance of tobacco suspension cell culture by the action of the H_2_S donor NaHS was eliminated by various calcium and calmodulin antagonists (Li et al., 2012). These results are consistent with the inhibition by calcium antagonists of the effect of hydrogen sulfide on the heat resistance of wheat coleoptiles and the activity of antioxidant enzymes (Kolupaev et al., 2017).

ROS and gasotransmitters are capable of both stimulating and inhibiting each other’s effects. A direct post-translational modification of key enzymes involved in regulation of redox homeostasis – NADPH oxidase, ascorbate peroxidase, catalase, and probably several others – which, as already noted, can be positive and negative, may be a component of hydrogen sulfide and nitric oxide competitive action (Aroca et al., 2015; Correa-Aragunde et al., 2015; Corpas et al., 2019a). In turn, ROS can be inducers of NO formation with the participation of nitrate reductase (Dubovskaya et al., 2011) and H_2_S synthesis under the influence of L/D-cysteine desulfhydrase (Shi et al., 2015). These gasotransmitters carry out both antagonism and synergy in a complex regulation of redox homeostasis by direct effect on the corresponding proteins as well as by forming signals that alter the state of transcription factors and expression of corresponding genes (**Figure 2**).

## Conclusion and prospects

Two key gasotransmitters, nitric oxide and hydrogen sulfide, are involved in the regulation of adaptive responses to low and high temperatures and are involved in a complex overall cellular signaling-regulatory network. Many of their effects are related to functional interactions with ROS, which are also key signaling mediators in formation of plant responses to extreme impacts. The close interaction between NO, H_2_S, and ROS is largely due to the presence of common targets for chemical modification. The main such target is the thiol groups of proteins. The probability of the type of protein modifications depends largely on the local concentrations of nitric oxide, hydrogen sulfide, hydrogen peroxide and other active molecules, as well as other conditions affecting these interactions (e.g. pH, redox potential, etc.). In other words, there may be competition between ROS, NO, and H_2_S for binding targets in proteins. At the same time, active concentrations of these molecules may change due to their direct chemical interaction with each other.

However, in addition to direct modifications of protein molecules, ROS, NO, and H_2_S are involved in complex processes of signal formation and transmission to the genetic apparatus. Modifications to individual protein molecules can have both immediate and long-term effects, which can be directed in different ways. For example, inhibition of individual enzymes by the direct NO or H_2_S action can result in the formation of a signal that induces gene expression of these enzymes. The most obvious example would be the induction of antioxidant enzymes gene expression involving ROS accumulated when these enzymes are inhibited by nitric oxide or hydrogen sulfide. The possibility of post-translational modifications by nitric oxide and hydrogen sulfide of key antioxidant enzymes – ascorbate peroxidase, catalase, SOD, and some others – has now been studied in detail (Begara-Morales et al., 2014; Arora and Bhatla, 2015; Correa-Aragunde et al., 2015; Corpas et al., 2019b). These modifications can either increase or decrease activity of these enzymes, which in turn leads to changes in cellular redox homeostasis and formation of signals involving ROS. NO and H_2_S are also known to modulate key ROS-generating enzymes, NADPH oxidase and certain forms of peroxidases (Clark et al., 2000; Arora et al., 2016; Shen et al., 2020). Thus, the above examples in themselves demonstrate the close and complex interaction of gasotransmitters with ROS. In turn, ROS and gasotransmitters realize a significant part of their signaling potential with calcium as a universal secondary messenger. As noted, direct and indirect effects of ROS, NO, and H_2_S on the calcium channel status and cytosolic calcium content are possible.

Signals involving ROS, NO, H_2_S, and Ca^2+^ are critical for plant adaptation to extreme temperatures. As already noted, they affect the state and expression of genes of transcription factors important for adaptation to high and low temperatures. NO, H_2_S, and ROS are involved in the activation of gene expression of antioxidant enzymes, enzymes of synthesis of proline, soluble carbohydrates, phenolic compounds, polyamines, and many other low-molecular-weight compounds that exhibit membrane-protective, chaperone, and antioxidant effects in plant cells. Although these effects have been studied for quite a long time, and are sometimes perceived as obvious, we are still far from a full understanding of the mechanisms of NO and H_2_S involvement in the regulation of adaptive responses. Suffice it to mention some of the paradoxical effects discovered in recent years and discussed in this review. In particular, the effect of negative nitric oxide regulation of plant adaptation to low temperatures and drought manifested in increased resistance in mutants of nitrate reductase genes (Costa-Broseta et al., 2018; Zhou et al., 2021). Apparently, the effects of increased antioxidant enzyme activity under the influence of scavengers and inhibitors of nitric oxide synthesis seem to be of the same series (Talukdar, 2013; Karpets et al., 2015b). A possible cause of these phenomena could be the negative effect of excess NO on plant cells (nitrosative stress). On the other hand, increased resistance in mutants defective in NO synthesis or increased plant resistance to stressors in the presence of NO antagonists may be due to the triggering of alternative signaling mechanisms which ensure successful activation of defense responses but are not seen in the presence of ‘basic’ NO signaling.

While the NO and ROS involvement in the formation of plant stress-protective reactions is complicated by the possibility of nitrosative and/or oxidative stress in excess of these compounds, the activation of plant defense reactions by the hydrogen sulfide action seems more unambiguous. However, this compound is also highly toxic. Suffice it to mention the well-known severe inhibition of heme-containing enzymes by hydrogen sulfide. In general, the effects of hydrogen sulfide in plant cells are much less studied compared to NO. It is worth mentioning the very contradictory information on the H_2_S effect on state of stomata. It seems that the influence of hydrogen sulfide on physiological processes depends very much on the different types of its interaction with reactive nitrogen species (direct interaction, competition for protein targets, influence on each other’s synthesis, and involvement in hormonal signaling). Many aspects of this interaction remain unexplored. Meanwhile, works showing a synergistic stress-protective effect on plants when they are treated simultaneously with exogenous NO and H_2_S indicate additional possibilities for the practical use of gasotransmitter donors in crop production. Another understudied application may be the use of endogenous gasotransmitter assays to test the resistance of breeding material. So far, such studies have been sporadic, focusing only on nitric oxide (Zhang et al., 2019). All in all, there is no doubt that the gasotransmitters NO and H_2_S have great potential for developing new techniques to practically increase the resistance of cultivated plants to temperature and other environmental stresses.

## Author contributions

YK: Writing original draft. AY: review and editing. TY: review and editing, YB: Review, editing and supervision. All authors contributed to the article and approved the submitted version.
